# Comment on An evaluation of the West of Scotland in-programme Chief Resident role^⁎^

**DOI:** 10.1016/j.fhj.2024.100139

**Published:** 2024-05-04

**Authors:** Anthony W. Martinelli

**Affiliations:** Cambridge Institute of Therapeutic Immunology & Infectious Disease (CITIID), Jeffrey Cheah Biomedical Centre, Department of Medicine, University of Cambridge, Cambridge CB2 0AW, UK

Editor – the West of Scotland in-programme Chief Resident role evaluated in this issue has clearly driven improvements in local services and resulted in professional development for the Chief Residents themselves, but I am concerned that such responsibilities were seemingly expected to be delivered in addition to full-time clinical commitments.^1^ In much the same way that consultants are allocated contractual time for significant responsibilities outside of direct clinical care, it is surely now right that the same becomes the case for non-consultant doctors? The article correctly highlights the benefits of quality improvement projects (QIPs) originating from ‘junior’ clinical staff, but protected time to undertake this would clearly reduce the risk of burnout, which was highlighted as a concern by some participants in this programme.

Clinical leadership schemes remain a popular way of developing a skillset in service improvement, but the most well-established of these all include specific, ring-fenced time during which these activities can be undertaken.^2^ The RCP's flagship Chief Registrar programme includes 40–50% protected non-clinical time and the majority of trainees pursue these posts whilst remaining in-programme.^3^ More recently, changing expectations of career structures and recruitment difficulties have resulted in the development of Flexible Portfolio Training, with 20% per week protected time for activities including clinical service improvement.^4^

Even outside of formal leadership schemes, doctors in postgraduate training should now be allocated protected ‘self-development time’, during which projects, including QIPs, can be undertaken. This should already be incorporated into rotas for doctors on the foundation programme (2 hours per week) and has also recently been advised for Internal Medicine Trainees (1 day per month in IMT1/2 and 2 days per month for IMT3s) and Higher Specialty Trainees (2 days per month).^5^, ^6^, ^7^ As the RCP Trainees Committee, we are strongly supportive of this protected time and will continue to encourage NHS organisations to adhere to this guidance. Importantly, given that training is competency-based, such protected time need not extend progression. We anticipate that giving trainees time to participate in these activities will result in service benefits for the NHS both in the present and the future, with doctors instituting workplace improvements now and gaining the meaningful experience in leadership which will help drive positive change in years to come.

Anthony W. Martinelli

Eastern Representative, RCP London Trainees Committee

## Declaration of competing interests

The authors declare that they have no known competing financial interests or personal relationships that could have appeared to influence the work reported in this paper.

## References

[1] Robertson C, Manners R, Bingham K, French H, Porteous KY, Oliver S (2024). An evaluation of the West of Scotland in-programme Chief Resident role. Future Healthcare J.

[2] Grote H, Smith J, Little J, Horridge M (2019). Clinical leadership fellow schemes for junior doctors: a brief overview of available schemes and how to apply. Future Healthc J.

[3] Royal College of Physicians. Chief registrar programme. [cited 2024 Feb 29]. Available from: https://www.rcplondon.ac.uk/projects/chief-registrar-programme.

[4] NHS England Workforce, Training and Education. Flexible portfolio training. [cited 2024 Feb 29]. Available from: https://www.hee.nhs.uk/our-work/doctors-training/flexible-portfolio-training.

[5] JRCPTB. Time for self-development in internal medicine training stage 1. 2022.

[6] NHS Employers. Foundation programme review - self development time. 2024 [cited 2024 Mar 1]. Available from: https://www.nhsemployers.org/articles/foundation-programme-review-self-development-time.

[7] JRCPTB. Guidance re self-development time for physicians in higher specialty training [Internet]. 2024. Available from: https://www.thefederation.uk/sites/default/files/2024-03/Guidance%20re%20self-development%20time%20for%20physicians%20in%20higher%20specialty%20training.pdf.

